# Prevalence and Recovery of Arrhythmia‐Induced Cardiomyopathy in Patients With Newly Diagnosed Heart Failure Using a Wearable Defibrillator: A Real‐World Cohort Study

**DOI:** 10.1111/jce.70366

**Published:** 2026-05-15

**Authors:** Joerg Yogarajah, Jana Dannebaum, Ahmed Halim, Jamschid Sedighi, Matthias Mensch, Malte Kuniss, Thomas Neumann, Andreas Rieth, Julia Treiber, Samuel Sossalla, Andreas Hain

**Affiliations:** ^1^ Department of Cardiology, Kerckhoff Heart and Thorax Center, Campus Kerckhoff Justus Liebig University Giessen Bad Nauheim Germany; ^2^ Department of Cardiology, Medical Clinic I Justus Liebig University Giessen Giessen Germany; ^3^ Cardio‐Pulmonary Institute (CPI), Excellence Cluster Justus‐Liebig‐University Giessen Bad Nauheim Germany

**Keywords:** arrhythmia‐induced cardiomyopathy, atrial fibrillation, heart failure, implantable cardioverter defibrillator, wearable cardioverter defibrillator

## Abstract

**Background:**

Arrhythmia‐induced cardiomyopathy (AIC) is a potentially reversible cause of heart failure triggered by different sustained arrhythmias, but data on its real‐world prevalence, recognition, and outcomes are limited.

**Objective:**

To assess frequency, predictors, and outcomes of AIC in patients with newly diagnosed left ventricular systolic dysfunction (LVSD) and concurrent arrhythmia undergoing early rhythm control with a wearable cardioverter‐defibrillator (WCD).

**Methods:**

Among 780 WCD‐treated patients (2017–2023), those with newly diagnosed idiopathic LVSD (LVEF < 35%) and persistent arrhythmia (atrial fibrillation/flutter or > 20% ventricular ectopy) were included. Diagnostic workup comprised echocardiography, coronary angiography, and cardiac MRI. Effective rhythm control was achieved via cardioversion, antiarrhythmic drugs, and/or ablation. Follow‐up was up to 6 months. AIC was defined as LVEF improvement > 15% with restored sinus rhythm or suppressed ventricular ectopy.

**Results:**

Of 780 WCD patients, 142 (18.2%) had LVSD with arrhythmia; 74 idiopathic cases were analyzed. At a mean follow‐up of 138 days (4.5 months), 54 patients (73%) maintained sinus rhythm or suppressed ventricular ectopy after rhythm control, of whom 32 (59.3%) fulfilled AIC criteria. LVEF improved from 28% to 43% in AIC; 56.3% of AIC patients fully recovered (> 50%). Non‐AIC patients had larger LV dimensions, lower heart rates, more mitral regurgitation, and a non‐significant trend toward more frequent late gadolinium enhancement (*p* = 0.073). No parameter reliably predicted AIC; LVEF < 25% predicted lack of full recovery (*p* = 0.040). No appropriate WCD shocks occurred. ICD indication decreased from 100% to 17% post‐therapy in AIC and Non‐AIC patients (3.1% vs*.* 36.4%, *p* = 0.001).

**Conclusions:**

AIC related to atrial fibrillation, flutter, or frequent ventricular ectopy is more prevalent and reversible than often recognized. Early rhythm control under WCD protection, supported by comprehensive diagnostics, allows identification of reversible LVSD and may help prevent unnecessary ICD implantation.

## Graphical abstract

This graphical abstract summarizes the study design and main findings in patients with newly diagnosed left ventricular systolic dysfunction and concomitant arrhythmia undergoing early rhythm control under wearable cardioverter‐defibrillator protection. Arrhythmia‐induced cardiomyopathy was identified in a substantial proportion of patients and was associated with significant recovery of left ventricular function, resulting in a marked reduction in implantable cardioverter‐defibrillator indications.

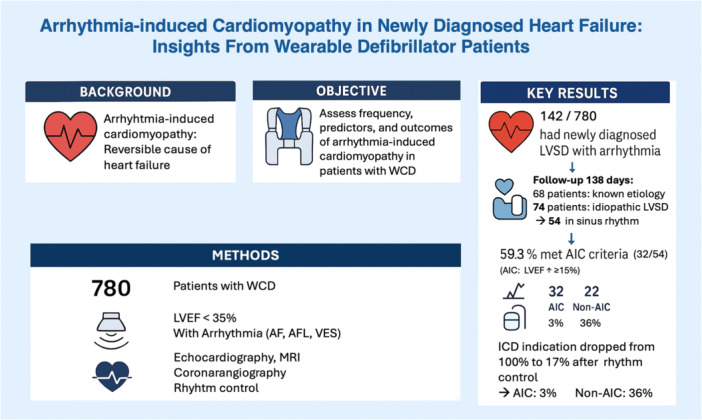

**Abbrevations**
AF: atrial fibrillation
AFL: atrial flutter
AIC: arrhythmia‐induced cardiomyopathy
DCM: dilated cardiomyopathy
ECG: electrocardiogram
ICD: implantable cardioverter‐defibrillator
LA: left atrium
LGE: late gadolinium enhancement
LVEF: left ventricular ejection fraction
LVSD: left ventricular systolic dysfunction
MRI: magnetic resonance imaging
NYHA: New York Heart Association
PVC: premature ventricular contractions
SCD: sudden cardiac death
VF: ventricular fibrillation
VT: ventricular tachycardia
WCD: wearable cardioverter‐defibrillator

## 1 Introduction

Arrhythmia‐induced cardiomyopathy (AIC) represents a distinct subset of dilated cardiomyopathies (DCM) and is clinically characterized by impaired left ventricular function (left ventricular systolic dysfunction, LVSD) secondary to an underlying arrhythmia [1, 2, 3, 4]. A hallmark feature of this condition is the partial or complete reversibility of left ventricular function following effective arrhythmia management. AIC can be caused by several types of arrhythmias, most commonly atrial fibrillation (AF), atrial flutter (AFL), or premature ventricular contractions (PVC) [3]. Importantly, AF/AFL may lead to AIC even in the absence of tachycardia [1, 5, 6].

Limited data exist on the prevalence of AIC, particularly in patients with severely reduced left ventricular ejection fraction (LVEF < 35%). Recent clinical data suggest that AIC is an underrecognized yet significant and reversible cause of heart failure, despite its high prevalence [6, 7]. This underestimation is largely attributable to the challenges in establishing the diagnosis. AIC is typically diagnosed retrospectively, with confirmation is possible only after a time period of appropriate therapy [4]. Consequently, in patients presenting with LVSD and concurrent arrhythmia, it remains challenging to distinguish between idiopathic cardiomyopathy with secondary arrhythmia and primary AIC at the initial evaluation. This is the chicken‐egg dilemma [2, 8, 9].

Patients with reduced LVEF are often at increased risk for life‐threatening ventricular arrhythmias and sudden cardiac death (SCD) [10, 11]. In these high‐risk individuals, wearable cardioverter‐defibrillators (WCDs) serve as a vital bridge therapy, providing protection against SCD while recovery of LVEF [12]. In AIC, the risk of ventricular arrhythmias and SCD is uncertain, as the diagnosis is rarely evident at initial presentation. This uncertainty highlights the role of the WCD as a bridge until reversibility of LV dysfunction can be established, potentially avoiding unnecessary ICD implantation in patients with reversible disease [13, 14, 15].

Despite their growing use, the prevalence and clinical trajectory of AIC in patients with idiopathic cardiomyopathy utilizing WCDs remain underexplored.

The aim of this study was to assess the prevalence, predictors, and outcome of AIC in patients with unexplained LVSD, and coexisting arrhythmias treated with WCDs, following a comprehensive diagnostic work‐up and exclusion of other potential etiologies. By exploring the role of WCDs in preventing adverse outcomes, supporting recovery of cardiac function, and guiding arrhythmia management, we seek to enhance the understanding of optimal care strategies for this unique and high‐risk patient population.

## 2 Methods

### 2.1 Study Population and Patient Selection

This retrospective observational cohort study included consecutive patients at high risk for life‐threatening ventricular arrhythmias, who were treated with a WCD at the Kerckhoff Heart Center in Bad Nauheim between 2017 and 2023. All participants were equipped with the LifeVest system (Zoll, Pittsburgh, USA).

Patients presenting with newly diagnosed LVSD characterized by a LVEF < 35% in the context of arrhythmias such as AF, AFL, or PVC burden > 20% as potential AIC candidates underwent comprehensive diagnostic evaluations, including 12‐lead ECG, echocardiography, coronary angiography, and cardiac magnetic resonance imaging (MRI).

Follow‐up visits were scheduled at 1, 2, and up to 6 months after WCD initiation. At each follow‐up, 12‐lead ECG and echocardiography was performed to assess LVEF recovery. In addition, heart failure medications were reviewed, adjusted, and optimized adhering to contemporary guidelines. Data were also collected from the Zoll LifeVest Network. At the end of the follow‐up period, patients were classified into two groups: idiopathic cardiomyopathy and LVSD with an identifiable etiology (e.g., valvular heart disease or myocarditis).

Patients with idiopathic LVSD who achieved restored sinus rhythm during follow‐up were further subdivided into AIC or non‐AIC based on treatment response. AIC was defined as an improvement in LVEF > 15% during follow‐up, while patients who exhibited persistent arrhythmias despite treatment were classified as non‐responders.

This study was approved by the Ethics Committee of Justus Liebig University of Giessen (Approval No. AZ 134/25) and adhered to the principles outlined in the Declaration of Helsinki. All patients were thoroughly informed about the WCD prior to participation.

### 2.2 Wearable Defibrillator

A commercially available WCD was used in this study (Life Vest system; Zoll, Pittsburgh, USA). The system comprises a vest with three self‐gelling defibrillation electrodes (two posterior, one anterior) and four ECG electrodes connected to a monitor. It continuously analyzes the ECG and can deliver up to five biphasic shocks (max. 150 J) in a posterior–anterior configuration [16]. Upon arrhythmia detection, based on heart rate, rhythm morphology, and event duration, the device triggers an escalating alert sequence (vibration, audible alarms). Patients can abort shocks by pressing response buttons during a ≥ 25‐s warning phase. Default detection thresholds are 150 bpm for ventricular tachycardia (VT) and 200 bpm for ventricular fibrillation (VF). The WCD also detects severe bradycardia or asystole, prompting voice‐guided emergency instructions and ECG recording.

### 2.3 Rhythm Control Strategy

Patients were treated with various rhythm control strategies, including electrical cardioversion, antiarrhythmic medication, and catheter ablation. Interventions for rhythm control were based on physician judgment and patient indication; multiple strategies could be applied.

### 2.4 Endpoints

The primary endpoints of this study were the prevalence of AIC and recovery from LVSD within this patient cohort. Secondary endpoints focused on identifying early predictors of AIC, including echocardiographic parameters (left atrial area, left ventricular end‐diastolic diameter [LVEDD], left ventricular end‐systolic diameter [LVESD], and mitral regurgitation), biomarkers such as NT‐proBNP, the presence of late gadolinium enhancement (LGE) on cardiac MRI, and New York Heart Association (NYHA) functional class. Further secondary endpoints were number of appropriate shocks, ICD‐implantations, and mortality at end of follow‐up. ICD implantations were performed for primary prevention according to guidelines, based on LVEF < 35% despite optimal medical therapy [17].

### 2.5 Statistical Analysis

Variables were expressed as mean ± standard deviation (SD) or median with interquartile range (IQR), as appropriate. Categorical variables were reported as absolute numbers and percentages. Group comparisons were performed using 2‐sample *t*‐tests for normally distributed continuous variables, Mann–Whitney *U* tests for non‐normally distributed variables, and chi‐squared tests for categorical data. Multivariable logistic regression was used to identify predictors of AIC diagnosis and full LVEF recovery. Model diagnostics included tests for variance, goodness‐of‐fit, and calculation of odds ratios with 95% confidence intervals. All analyses were conducted using Microsoft Excel and Minitab.

## 3 Results

### 3.1 Study Population and AIC Prevalence

Between September 2017 and June 2023, 780 consecutive patients with newly diagnosed LVSD who received a WCD were screened. Among them, 142 patients presented with a first diagnosis of LVSD in the presence of arrhythmia, including AF, AFL, or frequent VES (burden > 20%). The median follow‐up was 138 ± 39 days (≈4.5 months).

**Figure 1:**
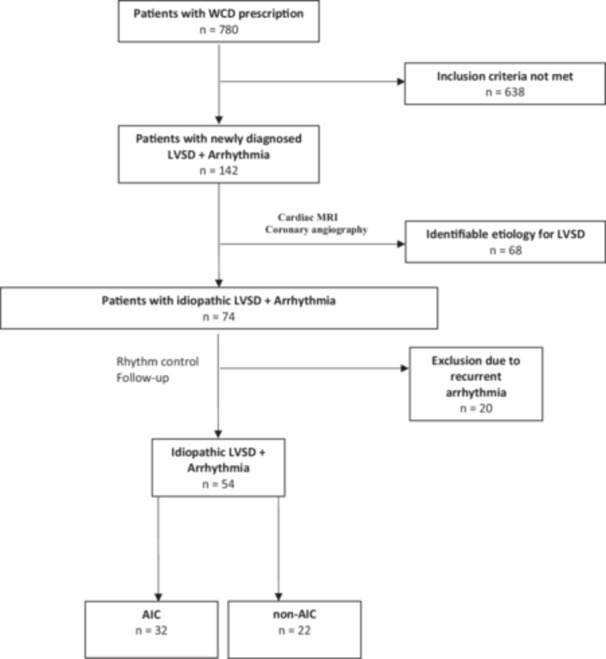
Flow chart—Diagnosis of AIC. AIC, arrhythmia induced cardiomyopathy; LVSD, left ventricular systolic dysfunction; MRI, magnetic resonance imaging; WCD, wearable defibrillator converter.

**Figure 2:**
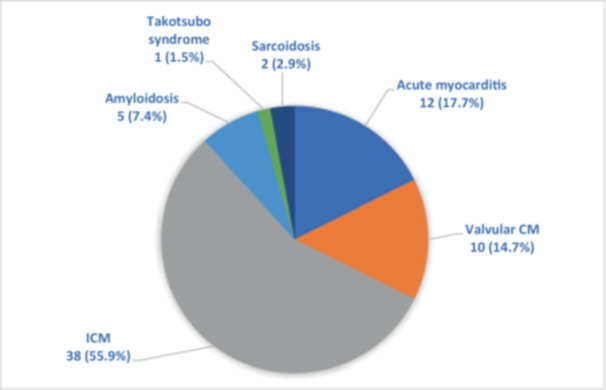
Identifiable etiology for left ventricular systolic dysfunction. CM, cardiomyopathy; ICM, ischemic cardiomyopathy.

Of these, 68 patients had an identifiable etiology of LVSD, while 74 patients were classified as having idiopathic LVSD (Figure 1). The known etiologies included ischemic cardiomyopathy (*n* = 38), valvular cardiomyopathy (*n* = 10), myocarditis (*n* = 12), cardiac sarcoidosis (*n* = 2), takotsubo cardiomyopathy (*n* = 1), and cardiac amyloidosis (*n* = 5). This is shown in Figure 2.

At follow‐up, 20 of the 74 idiopathic LVSD patients (27%) remained in arrhythmia and were categorized as rhythm control non‐responders. Among the 54 patients who achieved sinus rhythm, 32 (59%) fulfilled criteria for AIC, whereas 22 patients (41%) were classified as non‐AIC.

### 3.2 Baseline Characteristics

Baseline demographic and clinical characteristics were largely comparable between the AIC and non‐AIC groups (Table 1). Mean age, sex distribution, BMI, and prevalence of comorbidities such as diabetes and hypertension showed no significant differences. A history of smoking was more frequent in non‐AIC patients (45.5% vs. 18.8%, *p* = 0.037). AF was the predominant arrhythmia in both groups (81.3% AIC vs. 95.5% non‐AIC), followed by AFL. Ventricular ectopy (> 20%) was rare overall but present only in AIC patients (9.4%).

**Table 1:** Baseline characteristics of the study population.

Baseline characteristics	Total *N* = 54	AIC *N* = 32	Non‐AIC *N* = 22	*p*‐value
**Demographics and comorbidities**				
Age (years), mean ± SD	62.0 ± 12.0	62.2 ± 11.7	61.6 ± 12.7	0.853
Female sex, *n* (%)	6 (11.1)	3 (9.4)	3 (13.6)	0.362
Height (cm), mean ± SD	178.3 ± 8.3	178.9 ± 7.9	177.5 ± 8.9	0.579
Weight (kg), mean ± SD	96.6 ± 25.5	97.5 ± 27.7	95.1 ± 22.4	0.721
BMI (kg/m^2^), mean ± SD	30.3 ± 6.9	30.5 ± 7.7	30.0 ± 6.0	0.775
Smoker (ever), *n* (%)	16 (29.6)	6 (18.8)	10 (45.5)	0.037
Diabetes mellitus type 2, *n* (%)	7 (13.0)	3 (9.4)	4 (18.2)	0.349
Hypertension, *n* (%)	27 (0.5)	15 (46.9)	12 (54.5)	0.583
**NYHA class (%)**				
I	24.1	15.6	38.1	0.062
II	25.9	33.3	23.8	0.456
III	37.0	44.4	38.1	0.648
IV	1.9	3.5	0	0.383
**ECG, echocardiography, and MRI**				
Baseline LVEF (%), median (IQR)	26 (21.9–32.5)	27.5 (18.0–32.5)	24 (22.5–31.4)	0.986
Left atrial diameter (cm^2^), mean ± SD	29.5 ± 6.3	29.5 ± 6,1	28.8 ± 6.2	0.744
LVEDD (mm), mean ± SD	60.5 ± 8.0	58.7 ± 7.6	63.1 ± 7.9	0.047
LVESD (mm), mean ± SD	49.9 ± 7.6	47.8 ± 7.2	52.9 ± 7.2	0.015
Mitral valve regurgitation, *n* (%)	47 (87)	31 (97)	16 (73)	0.012
Mitral valve regurgitation degree				
I (%)	70.73	70.4	62.5	0.547
II (%)	29.27	29.6	37.5	0.547
III (%)	0	0	0	—
NT‐pro BNP (pg/mL) (mean ± SD)	5471 ± 6576	6111 ± 7407	4629 ± 5370	0.446
Heart rate (bpm) (mean ± SD)	106 ± 30	116 ± 27	90 ± 28	0.002
QRS width (ms) (mean ± SD)	109 ± 28	102 ± 23	118 ± 32	0.069
LGE positive in MRI, *n* (%)	34 (62.9)	17 (53.1)	17 (77.3)	0.073
**Type of underlying arrhythmia**				
Atrial fibrillation, *n* (%)	47 (87.0)	26 (81.3)	21 (95.5)	0.131
Atrial flutter *n* (%)	4 (7.41)	3 (9.4)	1 (4.5)	0.503
VES, *n* (%)	3 (5.56)	3 (9.4)	0 (0)	0.143
**Heart failure drugs at baseline**				
Beta blocker, *n* (%)	53 (98.2)	32 (100)	21 (95.5)	0.233
MRA, *n* (%)	52 (96.3)	31 (96.9)	21 (95.5)	0.793
Diuretics, *n* (%)	41 (75.9)	24 (75.0)	17 (77.3)	0.852
SGLT‐2 inhibitors, *n* (%)	22 (40.7)	13 (40.6)	9 (40.9)	0.981
Entresto, *n* (%)	31 (57.4)	17 (53.1)	14 (63.6)	0.450
ACE‐Inhibitors, *n* (%)	19 (35.2)	11 (34.4)	8 (36.4)	0.882
ARB, *n* (%)	4 (7.4)	3 (9.4)	1 (4.5)	0.510

Abbreviations: ACE, angiotensin converting enzyme; AIC, arrhythmia induced cardiomyopathy; ARB, angiotensin‐receptor‐blocker; BMI, body mass index; ECG, electrocardiography; IQR, interquartile range; LGE, late gadolinium enhancement; LVEDD, left ventricular end‐diastolic diameter; LVEF, left ventricular ejection fraction; LVESD, left ventricular end‐systolic diameter; MRA, mineralocorticoid‐receptor antagonist; MRI, magnetic resonance imaging; NT‐pro BNP, N‐terminal pro‐B‐type natriuretic peptide; NYHA, New York Heart Association; SD, standard deviation; SGLT, sodium‐dependent glucose‐co‐transporter; VES, ventricular extrasystole.

**Figure 3:**
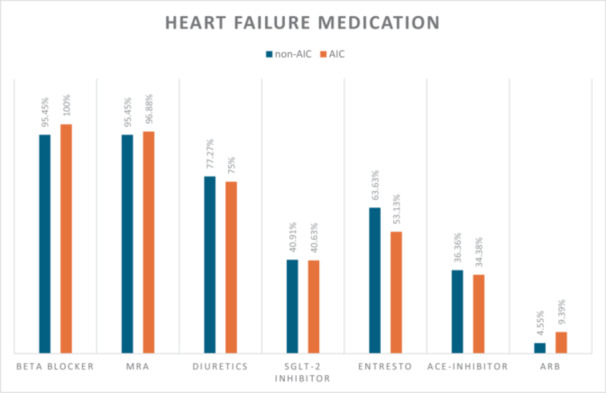
Heart failure medication prescribed to WCD patients. ACE, angiotensin‐converting enzyme; AIC, arrhythmia induced cardiomyopathy; ARB, angiotensin II receptor blocker; Entresto, Sacubitril/Valsartan; MRA, mineralocorticoid receptor antagonist.

Echocardiographic evaluation at WCD initiation revealed significantly larger LVEDD and LVESD in non‐AIC patients (*p* = 0.047 and *p* = 0.015, respectively), alongside a higher prevalence of mitral regurgitation (*p* = 0.012). Baseline heart rate was significantly higher in AIC patients (117 vs. 90 bpm, *p* = 0.002). There was a non‐significant trend toward more frequent ventricular late gadolinium enhancement (LGE) on cardiac MRI in the non‐AIC group (77.3% vs. 53.1%, *p* = 0.073). Heart failure medication usage at baseline was similar across both groups, with no significant differences (Table 1, Figure 3).

### 3.3 Rhythm Control

Rhythm control strategies included electrical cardioversion in 78.1% of AIC patients and 81.8% of non‐AIC patients (*p* = 0.743, Table 2). Antiarrhythmic medication was used in 46.9% and 50.0% respectively (*p* = 0.842), and catheter ablation in 53.1% of AIC patients versus 22.7% in non‐AIC (*p* = 0.027).

**Table 2:** Rhythm control.

Rhythm control strategy	AIC *N* = 32	Non‐AIC *N* = 22	*p*‐value
Electric cardioversion, *n* (%)	25 (78.1)	18 (81.8)	0.743
Antiarrhythmic drugs (class III), *n* (%)	15 (46.9)	11 (50.0)	0.824
Ablation, *n* (%)	17 (53.1)	5 (22.7)	0.027

Abbreviation: AIC, arrhythmia‐induced cardiomyopathy.

### 3.4 Left Ventricular Function Recovery

**Figure 4:**
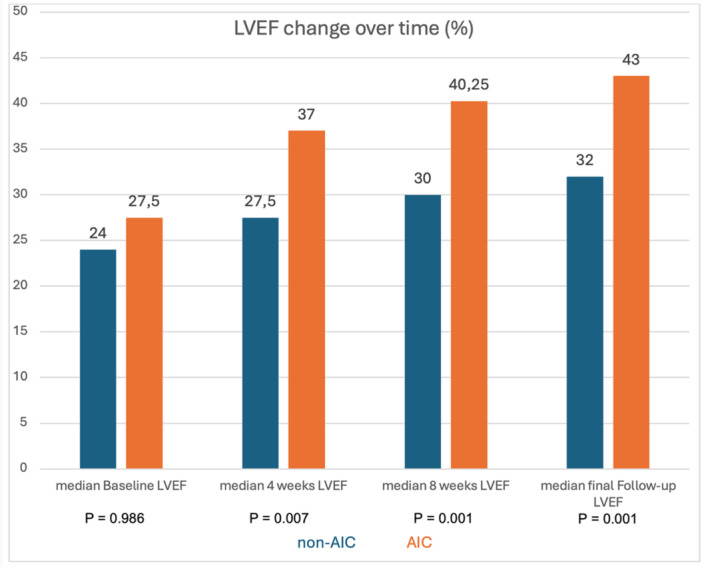
LVEF change over time in non‐AIC and AIC patients. Entresto, Sacubitril/Valsartan; LVEF, left ventricular ejection fraction.

Median LVEF improved significantly in AIC patients from 28% at baseline to 43% at follow‐up (*p* < 0.001), compared to a more modest increase in the non‐AIC group (24%–32%) (Figure 4). LVEF differences between groups were already apparent at 4 weeks (37% vs. 27.5%, *p* = 0.007) and widened further by 8 weeks (40% vs. 30%, *p* = 0.001). Full recovery of LVEF (> 50%) was achieved in 56.3% of AIC patients during follow‐up.

### 3.5 WCD Characteristics and ICD Implantation

Median WCD usage duration was 47 days (IQR: 28–61) in AIC patients and 59 days (IQR: 41–71) in non‐AIC patients (*p* = 0.125). Daily wear time was similar between groups (23.4 vs. 23.8 h/day; *p* = 0.17). No appropriate shocks were delivered by the WCD in either group. ICD implantation occurred in eight non‐AIC patients (36.4%) compared to only one patient (3.1%) in the AIC group (*p* = 0.001). Overall, the indication for ICD implantation according to current guidelines (LVEF < 35%) decreased from 100% to 17% (9/54) after rhythm control and optimized heart failure therapy in patients with idiopathic LVSD and concurrent arrhythmias. No deaths occurred in either the AIC or non‐AIC group during follow‐up.

### 3.6 Predictors of AIC and Full‐Recovery

In the logistic regression analysis for predicting AIC, none of the investigated variables reached statistical significance (Table 3). While QRS duration showed a trend toward significance (odds ratio [OR] 0.97; 95% confidence interval [CI] 0.94–1.00; *p* = 0.059), it did not meet the threshold. Other echocardiographic parameters, including LVEDD, LVESD, and LA size, as well as NT‐proBNP, baseline LVEF, and presence of mitral regurgitation or ventricular LGE on cardiac MRI, were not significantly associated with the occurrence of AIC.

**Table 3:** Predictors for arrhythmia‐induced cardiomyopathy.

Variables	Odds ratio (OR)	95% confidence interval	*p*‐value
NT‐proBNP (pg/mL)	1.00	0.99–1.00	0.671
QRS (ms)	0.97	0.94–1.00	0.059
LVEDD (mm)	1.08	0.79–1.47	0.599
LVESD (mm)	0.82	0.60–1.13	0.217
Left atrial area (cm²)	1.00	0.99–1.00	0.121
LVEF (%)	0.95	0.84–1.08	0.364
LV LGE	0.41	0.07–2.11	0.334
Mitral regurgitation	0.75	0.12–4.24	0.762
NYHA II	1.48	0.19–12.0	0.698
NYHA ≥ III	2.70	0.43–16.8	0.271

Abbreviations: LV LGE, Left ventricular late gadolinium enhancement; LVEDD, left ventricular end‐diastolic diameter; LVEF, left ventricular ejection fraction; LVESD, left ventricular end‐systolic diameter; NT‐pro BNP, N‐terminal pro‐B‐type natriuretic peptide; NYHA, New York Heart Association.

In contrast, when analyzing predictors of full LVEF recovery in AIC group (defined as LVEF > 50%), a baseline LVEF < 25% emerged as a significant negative predictor (OR 0.03; 95% CI 0.001–0.84; *p* = 0.040; Table 4). All other tested variables, including QRS duration, ventricular dimensions, NT‐proBNP, NYHA class, and presence of LGE, were not significantly associated with recovery. Notably, neither MRI findings nor atrial rhythm disturbances (e.g., AF or AFL) predicted recovery in this cohort.

**Table 4:** Predictors for full‐recovery in AIC group.

Variables	Odds ratio (OR)	95% confidence interval	*p*‐value
NT‐proBNP (pg/mL)	1.00	0.99–1.00	0.972
QRS (ms)	0.99	0.96–1.03	0.488
LVEDD (mm)	1.00	0.73–1.35	0.970
LVESD (mm)	0.89	0.62–1.29	0.531
LVEF (%)	0.84	0.64–1.10	0.235
Left atrial area (cm^2^)	1.00	0.99–1.00	0.956
NYHA II	1.80	0.20–16.1	0.604
NYHA ≥ III	2.05	0.25–17.2	0.487
LV LGE	2.34	0.26–20.5	0.445
Mitral regurgitation	1.82	0.22–15.4	0.580
LVEF < 30%	1.06	0.08–14.2	0.965
LVEF < 25%	0.03	0.001–0.84	0.040

Abbreviations: LV LGE, Left ventricular late gadolinium enhancement; LVEDD, left ventricular end‐diastolic diameter; LVEF, left ventricular ejection fraction; LVESD, left ventricular end‐systolic diameter; NT‐pro BNP, N‐terminal pro‐B‐type natriuretic peptide; NYHA, New York Heart Association.

## 4 Discussion

### 4.1 Main Findings

This study provides the first detailed characterization of a clearly defined population of AIC patients among WCD users in a real‐world setting, encompassing different underlying arrhythmias such as AF, AFL, and ventricular ectopy.

The key findings are as follows:

1.
**High prevalence of AIC:** AIC is relatively common in patients with idiopathic LVSD and AF, even in those with severely reduced LVEF (< 35%), highlighting the need for increased awareness and timely diagnosis in this population.
2.
**Predictors of AIC:** No significant predictors for the early identification of AIC were identified. While cardiac MRI and coronary angiography are essential for determining the underlying etiology, neither MRI nor echocardiographic parameters appeared to reliably predict the presence of AIC. LVEF < 25% was only negative predictor for full recovery of LVSD.
3.
**Role of the WCD:** The WCD proved to be a tool in bridging the critical period of left ventricular recovery, aiding in the diagnosis of AIC and guiding rhythm management strategies. No appropriate WCD therapies occurred, indicating a potential low risk of sudden cardiac death. Importantly, its use may have contributed to a significant reduction in ICD implantation rates.

### 4.2 Definition and Prevalence of AIC

To date, AIC remains a poorly defined clinical entity. In previous studies focusing on AIC, the definition varied considerably, with differing criteria LVEF reduction and inconsistent exclusion of other potential etiologies of non‐ischemic cardiomyopathy, such as inflammatory, infiltrative, or valvular cardiomyopathies, rather than strictly differentiating between non‐ischemic and ischemic cardiomyopathy [3, 6, 7, 18]. This heterogeneity is also reflected in a recent EHRA survey, in which respondents, primarily electrophysiology specialists, were asked about the cut‐off for LVEF recovery after rhythm control to diagnose AIC; reported thresholds ranged widely from 5% to 20% [19].

In our study, a 15% threshold for LVEF improvement was chosen to define AIC in patients with idiopathic LVSD, reflecting a clinically meaningful increase, in line with a previously published multicenter prospective trial that applied the same criterion [7]. Importantly, we also assessed the rate of full recovery in this patient cohort. A further strength of our study is the comprehensive diagnostic work‐up, providing a detailed and structured classification of non‐ischemic cardiomyopathies: all patients underwent both echocardiography and CMR. Detailed diagnostics enabled the identification of diverse underlying etiologies of LVSD, including myocarditis, cardiac amyloidosis, and sarcoidosis.

The prevalence of AIC in our study was relatively high at 59%, aligning with findings from previous studies reporting rates between 50% and 82% [6, 7, 20, 21, 22, 23]. Schach et al. reported a high prevalence of 82%, using similar AIC criteria. However, it is important to consider that patients in our cohort had lower baseline LVEF (AIC: 27.5% vs. non‐AIC: 24%), due to the selection criteria for WCD use. This likely reflects a population with more advanced heart failure, which may partly explain the slightly lower AIC prevalence in our study, as AIC tends to be less common in late‐stage LV dysfunction. Additionally, 20 out of 74 patients (27%) were non‐responders, which could suggest an underestimation of AIC prevalence due to arrhythmia recurrence or incomplete rhythm control during follow‐up.

### 4.3 Recovery of LVSD

The reported timeframe for AIC development (days to 6 months) aligns with our mean follow‐up of 138 ± 39 days, supporting its adequacy for assessing reversibility [3, 7, 15, 20, 22].

Our findings demonstrate a significantly greater improvement in LVEF among AIC patients compared to non‐AIC patients. While both groups showed LVEF improvement over time, the increase was more pronounced in the AIC group, rising from a median of 27.5% at baseline to 43% at follow‐up, compared to 24%–32% in non‐AIC patients. Importantly, this difference became evident early, already at 4 weeks (37% vs. 27.5%, *p* = 0.007), and continued to widen at 8 weeks (40% vs. 30%, *p* = 0.001), emphasizing the importance of close monitoring in the early phase.

Despite similar baseline NYHA functional class and comparable heart failure medication use between the groups, AIC patients experienced more rapid and more complete recovery. Full recovery of LVEF (> 50%) was achieved in over half of AIC patients (56.3%), highlighting the reversible nature of AIC when identified and treated promptly.

Medical heart failure therapy was comparable between both groups and likely contributed to LVEF improvement in all patients. However, the more pronounced recovery observed in the AIC group suggests that rhythm control was the primary driver of reverse remodeling. While heart failure therapy is known to improve LVEF over time, as shown in prior trials, the rapid and greater improvement seen in AIC patients underscores the dominant role of effective rhythm control in this population [7, 24, 25, 26]. In our study, rhythm control strategies were mainly based on electrical cardioversion and pharmacological treatment with antiarrhythmic agents. A higher proportion of AIC patients underwent catheter ablation, likely as a consequence of the established diagnosis of AIC and the clinical indication for early rhythm control [27]. This may, at least in part, explain the observed differences between the AIC and non‐AIC groups. Moreover, during the study period (2017–2023), the evidence base and clinical adoption of AF ablation in heart failure were more limited than today [6, 28, 29, 30].

### 4.4 Predictors of AIC and Full‐Recovery

In our study, no reliable predictors of AIC were identified. However, non‐AIC patients tended to have larger left ventricular dimensions, lower heart rates, and more frequent mitral regurgitation. Previous studies have suggested that smaller left ventricular diameters may help distinguish AIC from non‐AIC patients [7, 31, 32, 33]. However, our findings are consistent with those of Müller‐Edenborn et al., who also found no significant predictive parameters for AIC [15]. In our cohort, non‐AIC patients demonstrated a non‐significant trend toward more frequent LGE on cardiac MRI (*p* = 0.073), but LGE was not predictive of AIC. Data on left ventricular LGE are conflicting. While Schach et al. reported no differences in the presence or extent of LGE between AIC and non‐AIC patients, other studies have shown a positive association between LGE and irreversible myocardial damage, thus suggesting a lower likelihood of AIC [7, 34, 35]. In the CAMERA‐MRI study, a lower burden of LGE was identified as a potential marker for AIC, further emphasizing the potential role of myocardial fibrosis assessment in this context [6].

In addition, AIC patients appear to exhibit markedly lower levels of atrial septal fibrosis, as demonstrated in a subanalysis of DECAAF II trial [22].

The absence of identifiable predictors for AIC (including LGE in MRI) in our study may be explained by the advanced stage of LVSD at baseline (median LVEF 27.5% vs. 24%) and the relatively small number of patients with idiopathic LVSD (*n* = 54), which may have limited statistical power to detect subtle associations.

Notably, an LVEF < 25% was the only variable associated with incomplete recovery, serving as a negative predictor even among patients ultimately classified as AIC. This finding contrasts with a prior study that suggested lower baseline LVEF may be associated with greater relative improvement following arrhythmia treatment in AIC [36]. The discrepancy may, at least in part, be explained by the more advanced degree of LVSD in our cohort at baseline. This likely reflects irreversible structural remodeling, potentially driven by a high atrial fibrillation burden or delayed recognition of the arrhythmia as the primary etiology of cardiomyopathy [5, 37]. An additional possible explanation is that some patients in our cohort may have AIC superimposed on a previously unrecognized (“occult”) cardiomyopathy, which could limit the extent of functional recovery [9]. These findings underscore the potential prognostic relevance of baseline LVEF severity for long‐term systolic recovery, while no reliable baseline predictors for AIC could be identified in this dataset.

### 4.5 Role of WCD

A recent EHRA survey on AIC found that only 21% of responding centers currently consider the WCD as a therapeutic option in AIC patients, highlighting the need for greater awareness of its potential role in this context and supporting the relevance of our findings [19].

To our knowledge, only one previous study has specifically investigated the use of the WCD in patients with AIC [14]. That study primarily focused on suspected rather than confirmed AIC cases. In contrast, our study examined a clearly defined AIC population, based on retrospective diagnosis and a standardized threshold for left ventricular functional recovery, representing, to our knowledge, the largest AIC cohort among WCD users reported to date.

Erath et al. reported a suspected AIC prevalence of 15% (20/130) in their WCD cohort, with 13 (65%) of those patients showing an LVEF improvement greater than 10%, which represents a lower threshold than to the > 15% improvement criterion used in our analysis [14].

Interestingly, no episodes of VT and no shocks were documented in our AIC and Non‐AIC population during WCD time, suggesting a low risk of sudden arrhythmic death in our patient cohort. In comparison, Erath et al. reported one episode of non‐sustained VT in the AIC cohort during WCD use.

However, the absence of appropriate WCD therapies in our cohort should be interpreted in the context of the distinct patient population studied. In contrast to prior studies in non‐ischemic cardiomyopathy (NICM), our cohort comprised patients with carefully characterized idiopathic cardiomyopathy with and without AIC, based on systematic evaluation including cardiac magnetic resonance imaging and coronary angiography. This more specifically defined population may be associated with a lower intrinsic risk of ventricular arrhythmias. Given the heterogeneity of NICM populations, which may include patients with underlying structural myocardial disease and higher arrhythmic risk, direct comparison is limited. Previous studies have reported relevant rates of appropriate WCD therapies in NICM populations, including a cohort demonstrating both improvement in LVEF with prolonged WCD use and 12 appropriate shocks in 11 patients, as well as the recent SCD‐PROTECT Study, which also showed substantial rates of appropriate WCD interventions in both NICM and post‐myocardial infarction patients [38, 39].

ICD implantation was required in 8 non‐AIC patients (36.4%), whereas only one patient (3.1%) in the AIC group received an ICD (*p* = 0.001), demonstrating a significantly lower implantation rate among patients with AIC, a finding consistent with the observations by Erath et al. [14]. Müller‐Edenborn et al. also demonstrated that early rhythm control in patients with LVSD (LVEF < 40%) reduced the need for ICD implantation as primary prevention from 76% to 11% [15]. In our study, the marked reduction in ICD indication (from 100% to 17% in AIC and non‐AIC patients) following rhythm control highlights the central role of arrhythmia in the development of heart failure and underscores the therapeutic importance of early rhythm stabilization.

Nevertheless, it remains unclear whether patients with AIC are at a lower or comparable risk of SCD than non‐AIC patients, as robust long‐term data on arrhythmic risk in this population are still lacking [13, 14]. While no appropriate WCD therapies were observed in our cohort, this finding should be interpreted in the context of the specifically defined AIC population and limited follow‐up. Overall, our data suggest that patients with idiopathic LVSD and coexisting arrhythmia who receive early rhythm control alongside heart failure drug therapy exhibit a low risk of sudden cardiac death while wearing a WCD, supporting the potential benefit of an early, combined treatment strategy.

### 4.6 Clinical Implications

To our knowledge, this study offers the first comprehensive characterization of AIC among WCD users in a real‐world clinical setting, revealing a notably high prevalence of AIC in patients with idiopathic LVSD and coexisting arrythmias, even with severely reduced LVEF. Importantly, this cohort comprised patients with various arrhythmia types—AF, AFL, and frequent PVC ‐ illustrating the heterogeneous arrhythmic triggers that can both cause and reverse LV dysfunction. The absence of reliable early predictors underscores the need for enhanced diagnostic strategies and early rhythm control. The WCD demonstrated critical value in managing the vulnerable recovery phase, facilitating AIC diagnosis, and potentially reducing unnecessary ICD implantations. No appropriate WCD shocks occurred, indicating a low SCD risk and supporting a conservative, rhythm‐focused approach to ICD decision‐making.

Prospective, multicenter studies are warranted to establish standardized diagnostic criteria, better define AIC prevalence at initial heart failure diagnosis or WCD use and identify predictors as well as SCD risk to guide personalized treatment and optimize outcomes.

### 4.7 Study Limitations

This study has some limitations. It is a retrospective, observational single‐center analysis with a relatively small sample size, which may limit generalizability.

Rhythm control strategies were not standardized due its retrospective design.

Rhythm status was assessed by serial 12‐lead ECGs and review of wearable cardioverter‐defibrillator data in case of device alerts or heart rate abnormalities. This may have underestimated short or paroxysmal atrial arrhythmias, particularly asymptomatic atrial fibrillation. However, no clinically relevant sustained arrhythmias were observed.

Medical heart failure therapy likely contributed to LVEF improvement, possibly leading to overestimation of AIC, though it remains an essential part of treatment. Moreover, no universally accepted diagnostic definition of AIC exists; although our definition aligns with previous studies, some degree of misclassification cannot be excluded.

## 5 Conclusion

AIC shows a high prevalence and represents a potentially reversible cause in patients with idiopathic LVSD, even at markedly reduced ejection fraction levels. Although no reliable early predictors could be identified, non‐AIC patients showed correlations with larger LV dimensions, lower heart rates, more mitral regurgitation, and a trend toward more frequent LGE. Importantly, early rhythm control facilitated substantial ventricular recovery in AIC, while no appropriate WCD therapies occurred, indicating a low short‐term risk of sudden cardiac death. These findings highlight the importance of comprehensive diagnostics, early rhythm stabilization with optimal medical heart failure therapy, and structured follow‐up to differentiate reversible AIC from underlying cardiomyopathy and to potentially prevent unnecessary ICD implantation.

## Ethics Statement

The study was performed in compliance with the principles outlined in the Declaration of Helsinki and approved by the ethics committee of Justus Liebig University of Giessen and registered under No. AZ 134/25.

## Consent

The authors have nothing to report.

## Conflicts of Interest

Andreas Hain received speaker fees from Zoll. Samuel Sossalla recieved speakers/consulting honoraria from Astra Zeneca, Novartis, Berlin‐Chemie, Bristol‐Myers‐Squibb, Boehringer Ingelheim, Lilly, Bayer, Pfizer. The remaining authors declare no conflicts of interest. S.S. is funded by the Deutsche Forschungsgemeinschaft (DFG) through the research grants 471241922 and 549060740, the project B10N of the Collaborative Research Center 1213‐Pulmonary Hypertension and Cor Pulmonale DFG, and the F. Thyssen Foundation (Az 10.19.2.026MN).

## Data Availability

The data that support the findings of this study are available from the corresponding author upon reasonable request.
